# ATP10A deficiency results in male-specific infertility in mice

**DOI:** 10.3389/fcell.2024.1310593

**Published:** 2024-02-13

**Authors:** Adriana C. Norris, Eugenia M. Yazlovitskaya, Tzushan Sharon Yang, Alex Mansueto, John M. Stafford, Todd R. Graham

**Affiliations:** ^1^ Department of Biological Sciences, Vanderbilt University, Nashville, TN, United States; ^2^ Division of Comparative Medicine, Department of Pathology, Microbiology and Immunology, Vanderbilt University Medical Center, Nashville, TN, United States; ^3^ Tennessee Valley Healthcare System, Nashville, TN, United States; ^4^ Division of Endocrinology, Diabetes and Metabolism, Vanderbilt University Medical Center, Nashville, TN, United States; ^5^ Department of Molecular Physiology and Biophysics, Vanderbilt University, Nashville, TN, United States

**Keywords:** infertility, male-specific infertility, fertility, P4-ATPase, flippase, knockout mouse models, phosphatidylcholine, glucosylceramide

## Abstract

Over 8% of couples worldwide are affected by infertility and nearly half of these cases are due to male-specific issues where the underlying cause is often unknown. Therefore, discovery of new genetic factors contributing to male-specific infertility in model organisms can enhance our understanding of the etiology of this disorder. Here we show that murine ATP10A, a phospholipid flippase, is highly expressed in male reproductive organs, specifically the testes and vas deferens. Therefore, we tested the influence of ATP10A on reproduction by examining fertility of *Atp10A* knockout mice. Our findings reveal that *Atp10A* deficiency leads to male-specific infertility, but does not perturb fertility in the females. The *Atp10A* deficient male mice exhibit smaller testes, reduced sperm count (oligozoospermia) and lower sperm motility (asthenozoospermia). Additionally, *Atp10A* deficient mice display testes and vas deferens histopathological abnormalities, as well as altered total and relative amounts of hormones associated with the hypothalamic-pituitary-gonadal axis. Surprisingly, circulating testosterone is elevated 2-fold in the *Atp10A* knockout mice while luteinizing hormone, follicle stimulating hormone, and inhibin B levels were not significantly different from WT littermates. The knockout mice also exhibit elevated levels of gonadotropin receptors and alterations to ERK, p38 MAPK, Akt, and cPLA_2_-dependent signaling in the testes. *Atp10A* was knocked out in the C57BL/6J background, which also carries an inactivating nonsense mutation in the closely related lipid flippase, *Atp10D.* We have corrected the *Atp10D* nonsense mutation using CRISPR/Cas9 and determined that loss of *Atp10A* alone is sufficient to cause infertility in male mice. Collectively, these findings highlight the critical role of ATP10A in male fertility in mice and provide valuable insights into the underlying molecular mechanisms.

## Introduction

Infertility remains a significant health problem that affects 8%–12% of couples worldwide and 40%–50% of cases are due to male-specific factors (Kumar and Singh, 2015). Male factor infertility is often due to poor semen quality; including low sperm count, reduced motility, and altered morphology. There are several potential causes of male infertility, such as hormonal imbalances, physical abnormalities, sexually transmitted diseases, lifestyle factors, and genetic factors (Babakhanzadeh et al., 2020). However, the cause of male factor infertility remains unknown in about 40% of patients (Winters and Walsh, 2014; Krausz and Riera-Escamilla, 2018). Therefore, the discovery of new genetic factors that result in male-specific infertility could help elucidate the molecular basis of this type of reproductive defect.

One potential genetic factor is *ATP10A*, a gene that encodes for a lipid translocating P4-ATPase. ATP10A translocates phosphatidylcholine (PC) and glucosylceramide (GlcCer) (Roland et al., 2019) across the plasma membrane and transcripts from this gene are expressed in several human (Consortium, 2013; Soraggi et al., 2021) and mouse tissues, (Wang et al., 2018; Wang et al., 2023) including reproductive organs. Additionally, the lipid substrates of ATP10A have established roles in fertility. PC is a major component of membranes in all cells and levels of this phospholipid and its acyl chain composition can substantially affect the function of the male reproductive tract (Mann, 1964; Mann and Lutwak-Mann, 1981; Lenzi et al., 1996; Lin et al., 2004). Furthermore, PC is an important component of the sperm plasma membrane and levels of this phospholipid can act as an indicator of sperm fertilization potential (Evans et al., 1980; Shan et al., 2021). Additionally, supplementing growth medium with PC expedites the *in vitro* development of human sperm; assessed by the molecular response of the sperm acrosome to progesterone (acrosomal responsiveness) (Cross, 1994). GlcCer metabolism has also been implicated in fertility; mice deficient for the non-lysosomal glucosylceramidase (*Gba2*), an enzyme that cleaves GlcCer into glucose and ceramide, exhibit GlcCer accumulation in the testes and impaired fertility associated with abnormal acrosomes and defective sperm mobility (Yildiz et al., 2006).

ATP10A has been shown to have a role in metabolism. A genome-wide association study reported a correlation between *Atp10A* variants and increased risk for developing insulin resistance (Irvin et al., 2011) and mice harboring large, irradiation-induced chromosomal deletions, that included *Atp10A,* exhibited worsened metabolic profiles (Dhar et al., 2004; Dhar et al., 2006). We recently produced an *Atp10A* exon 2 knockout (KO) mouse model and found that *Atp10A* ^*−/−*^ mice exhibit sex-specific diet-induced dyslipidemia (Norris et al., 2024). We found that the female KO mice fed a high fat diet display elevated levels of plasma cholesterol, triglycerides, and free fatty acids relative to wild-type (WT) littermates and this phenotype was not observed in the male KO mice. Interestingly, there is evidence indicating a connection between metabolic status and the expression of *ATP10A* in spermatozoa; indeed, type 2 diabetic patients exhibit differential DNA methylation patterns of *ATP10A* in human spermatozoa, compared to healthy individuals (Chen et al., 2020). However, the reproductive capability of the male *Atp10A* KO mice has not yet been assessed.

The mouse genome contains 15 P4-ATPase genes annotated *Atp8A1* to *Atp11C* and a few members of this protein family have been implicated in male fertility. *Atp8B1* is highly expressed in the acrosome region of sperm and disruption of this gene causes a modest reduction in male fertility; where morphologically normal and fully motile sperm are produced by the mutant males but the acrosome reaction *in vitro* is abrogated (Wang et al., 2004). The similar *Atp8B5* gene is also highly expressed during spermatogenesis although its impact on fertility is unknown (Xu et al., 2009). The *Atp10B* and *Atp10D* genes are highly homologous to *Atp10A* and encode PC/GlcCer (*Atp10B*) (Martin et al., 2020) and GlcCer (*Atp10D*) (Roland et al., 2019) translocases. Interestingly, the C57BL/6J inbred mouse line carries a naturally occurring nonsense mutation in the middle of the *Atp10D* open reading frame (Flamant et al., 2003). The truncated ATP10D protein in C57BL/6J mice lacks structurally essential components of the transporter and should be nonfunctional. The extent of functional redundancy, if any, between the *Atp10A* and *Atp10D* genes is unknown.

Given the transcription of *ATP10A* in male reproductive organs and its involvement in the translocation of bioactive lipids with roles in fertility; we examined fertility parameters in C57BL/6J mice lacking *Atp10A*. We also corrected the *Atp10D* nonsense codon in C57BL/6J mice to the wild-type glutamine codon to determine if *Atp10A* deficiency alone is sufficient to cause male-specific infertility.

## Results

### ATP10A is expressed in murine vas deferens and testes

**FIGURE 1:**
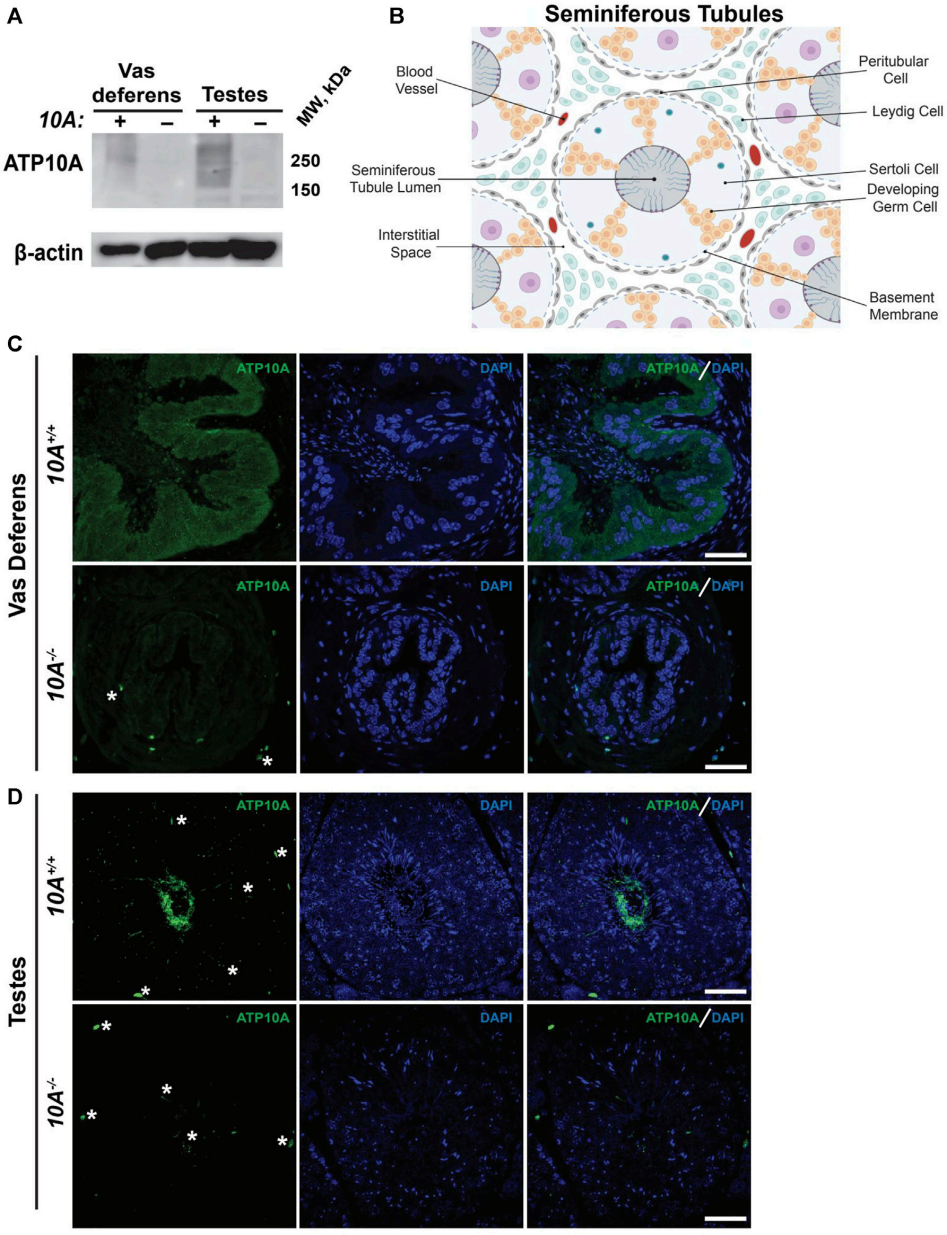
ATP10A is expressed in vas deferens and testes. **(A)** Total tissue lysates from vas deferens and testes from the *Atp10A* ^*+/+*^ *(10A*+) and *Atp10A* ^*−/−*^ *(10A*-) mice were subjected to Western immunoblot analysis for expression of ATP10A (100 µg total protein) using the anti-ATP10A antibody described in Supplementary Figure S1. β-actin served as a loading control. Note that ATP10A migrates as a high-molecular-weight smear. **(B)** Schematic of the seminiferous tubules as well as the cell types and blood vessels found in the interstitial space. Schematic adapted from Rato et al. (2003) and created using Biorender. com. **(C and D)** Representative immunofluorescent images of **(C)** vas deferens and **(D)** testes showing expression of ATP10A (green) and DAPI (blue). Scale bars = 40 µm. White asterisks indicate background staining (method for determining background staining is in Supplementary Figure S3 and Materials and Methods). (Vas deferens: *10A^+/+^* n = 1, *10A^−/−^* n = 2, Testes: *10A^+/+^* n = 2, *10A^−/−^* n = 2).

To begin to explore the potential role of ATP10A in fertility, we first tested if ATP10A protein is expressed in male reproductive organs. To do this, we produced anti-ATP10A antibodies to three different peptide sequences conserved between human and mouse ATP10A but not present in other P4-ATPases (Supplementary Figure S1A, B). We then tested the specificity of these different antibodies toward human ATP10A in HeLa cells overexpressing an HA-tagged ATP10A and found that the affinity purified antibody targeting amino acids 27–38 (labeled peptide 1) was specific for ATP10A (Supplementary Figure S1C, D). Note that ATP10A is a large, integral membrane protein that often aggregates when subjected to SDS-PAGE and migrates as a high molecular weight smear around 250 kDa. We then used immunoblot analysis to probe two male reproductive organs, the vas deferens, an epithelial and smooth muscle tubule, that transports sperm from the epididymis to the urethra during ejaculation, and the testes, for ATP10A expression (an image of these organs is provided in Supplementary Figure S2). We observed a high molecular weight smear around 250 kDa, specific for ATP10A, in the vas deferens and testes that was absent in *Atp10A* deficient mice (Figure 1A). Additionally, we observed a specific ATP10A-positive signal via immunofluorescence (IF) in WT mice that was absent in KO mice in the vas deferens (Figure 1C). We also observed a specific ATP10A-positive signal in the lumen of seminiferous tubules (the functional unit of the testes) where flagella of sperm are found at intermediate stages (VI-VIII) of the seminiferous epithelial cycle (Figures 1B,D, Supplementary Figure S3A, B). The structures stained with anti-ATP10A did not colocalize with peanut agglutinin lectin (PNA), which is an acrosome-specific marker of developing spermatids and mature sperm (average Manders’ Coefficient: 0.033, Supplementary Figure S3C). Taken together, ATP10A is expressed in the vas deferens and in the seminiferous tubule lumen.

### *Atp10A* deficiency results in a male-specific reduction in fertility, small testes, and low sperm count

**FIGURE 2:**
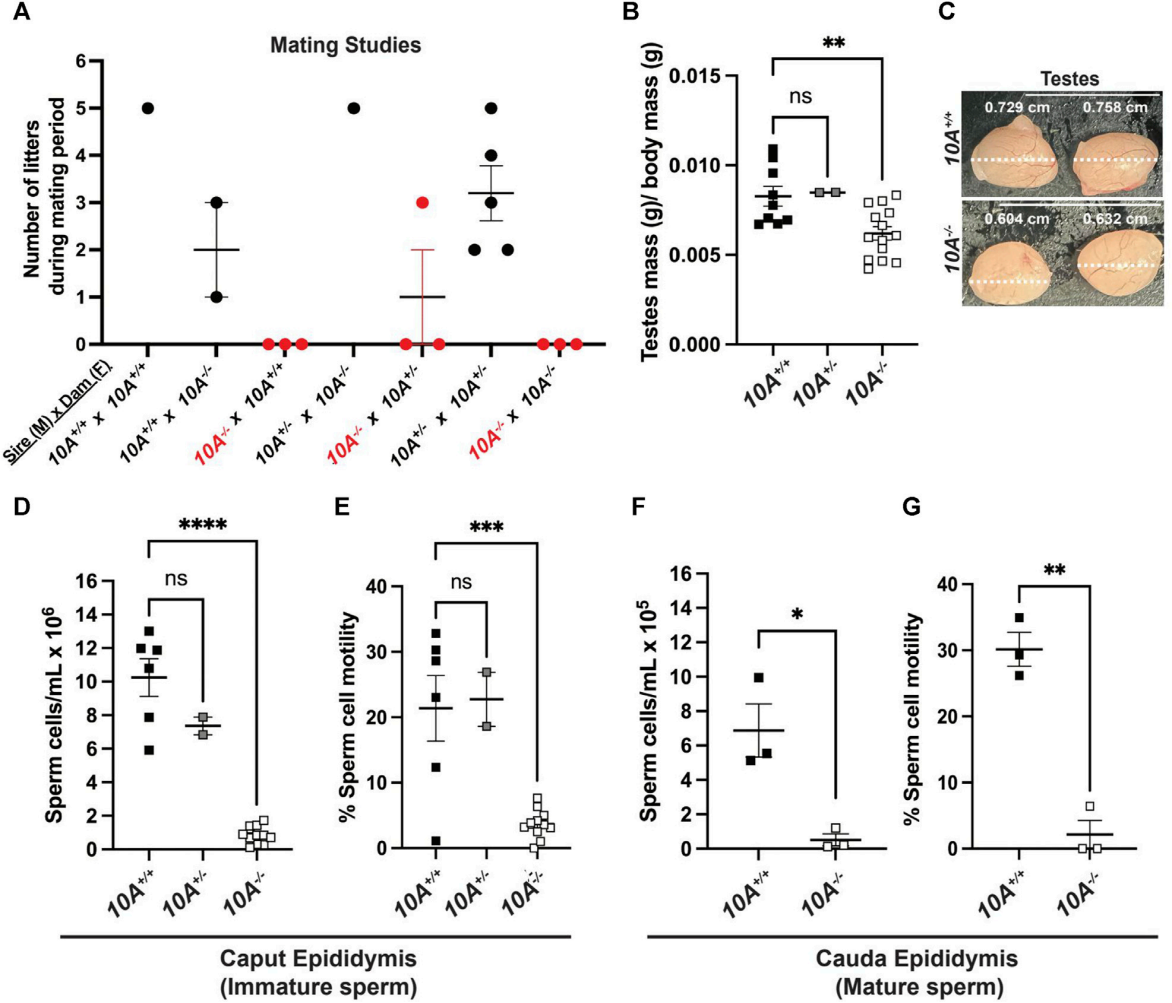
*Atp10A* deficiency results in male-specific infertility, smaller testes and reduced sperm count and motility. **(A)** Mating studies were done by housing pairs of mice together (without separating) for 6–21 weeks and recording the number of litters produced. (See Supplementary Table S1 for more details), **(B)** The wet mass of testes was measured in 8–19-week-old mice and was normalized to body mass of the mice, **p = 0.0060. (See Supplementary Table S1 for the unadjusted testes mass and body mass values). **(C)** Testes were visualized using a dissection scope and the width of the testes was measured using ImageJ. **(D–G)** Sperm cells collected from the **(D)** caput and **(F)** cauda epididymis were counted using a hemocytometer, ****p < 0.0001, *p = 0.0160. **(E and G)** % Sperm cell motility was measured using the equation: motile sperm/total sperm counted*,* ***p = 0.0003, **p = 0.0011. (B, D, **(E)** *p*-value by ordinary one-way ANOVA with Dunnett’s multiple comparison test, F,G: *p*-value by unpaired t-test). (B,D,E: *10A* ^*+/+*^ n = 9*, 10A* ^*+/−*^ n = 2*, 10A* ^*−/−*^ n = 14, F,G: *10A* ^*+/+*^ n = 3, *10A* ^*−/−*^ n = 3).

Given the expression of ATP10A in male reproductive organs, we next investigated whether the loss of *Atp10A* in mice would perturb fertility. To explore this, we housed male and female mice together for at most 21 weeks and recorded the number of litters they produced (Figure 2A) as well as the average number of pups born in each litter (Supplementary Table S1). We observed that the majority of pairs with *10A* ^*−/−*^ sires (a total of 9 *10A* ^*−/−*^ sires were examined) were unable to produce litters, with a single exception (see cross: *10A* ^*−/−*^ *(M) x 10A* ^*+/−*^ *(F),* Figure 2A), while pairs with *10A* ^*−/−*^ dams were able to produce litters. Additionally, the heterozygous mice (*10A* ^*+/−*^) that are used to breed the *10A* ^*+/+*^ and *10A* ^*−/−*^ experimental mice had an average of 3 litters during the mating period of approximately 12 weeks. Taken together, *Atp10A* deficiency results in a male-specific reduction in fertility.

We next measured the mass of testes, normalized to body mass of the mice, and found that *10A* ^*−/−*^ mice exhibited 1.2-fold lower average testes mass compared to *10A* ^*+/+*^ mice (Figure 2B) and also appeared visibly smaller (Figure 2C). To further probe the effects of *Atp10A* deficiency on male fertility we measured the number of sperm/mL in the caput epididymis, where immature sperm is stored, and cauda epididymis, where mature sperm is stored; as well as the motility of the sperm cells. We found that in the caput epididymis; *10A* ^*−/−*^ mice exhibit 12.2-fold and 8.7-fold fewer sperm/mL compared to *10A* ^*+/+*^ and *10A* ^*+/−*^ littermates, respectively (Figure 2D) and the percent of motile sperm from *10A* ^*−/−*^ mice was 5.8-fold and 6.2-fold lower compared to *10A* ^*+/+*^ and *10A* ^*+/−*^ mice, respectively (Figure 2E). In the cauda epididymis; *10A* ^*−/−*^ mice exhibit 13.4-fold fewer sperm/mL compared to *10A* ^*+/+*^ mice (Figure 2F). Additionally, the percentage of motile sperm from *10A* ^*−/−*^ mice was 14.2-fold lower compared to *10A* ^*+/+*^ mice (Figure 2G). Altogether, *Atp10A* deficiency results in small testes, oligozoospermia, and asthenozoospermia.

### Restoring *Atp10D* expression does not rescue the infertility phenotypes observed in *Atp10A* deficient mice

C57BL/6J mice have a naturally occurring premature stop codon in *Atp10D* (*10D*), a closely-related homolog of *Atp10A* (Flamant et al., 2003). Therefore, the genotypes of the mice used in this study were *10A* ^*+/+*^ *10D* ^*−/−*^ (WT) and *10A* ^*−/−*^ *10D* ^*−/−*^ (KO) and it was formally possible that the male infertility observed in the *10A* ^*−/−*^ *10D* ^*−/−*^ (KO) mice was caused by loss of both *Atp10A* and *Atp10D*. To determine if *Atp10A* deficiency was sufficient to cause this phenotype, we generated *10D* ^*+/+*^ mice via CRISPR-mediated repair of the nonsense mutation back to the wild-type glutamine codon and confirmed the genotype of the mice via sequencing (Supplementary Figure S4A). We found that male *10A* ^*−/−*^ *10D* ^*+/+*^ mice, were unable to produce litters (Supplementary Figure S4B), had significantly smaller testes (normalized to body mass) (Supplementary Figure S4C) as well as 16.2-fold fewer sperm/ml (Supplementary Figure S4D) and 4.1-fold less motile sperm (Supplementary Figure S4E), compared to *10A* ^*+/+*^ *10D* ^*+/+*^ mice. Therefore, ATP10A deficiency alone is sufficient to cause the male-specific infertility phenotypes.

### *Atp10A* deficient mice display testes and vas deferens pathologies

**FIGURE 3:**
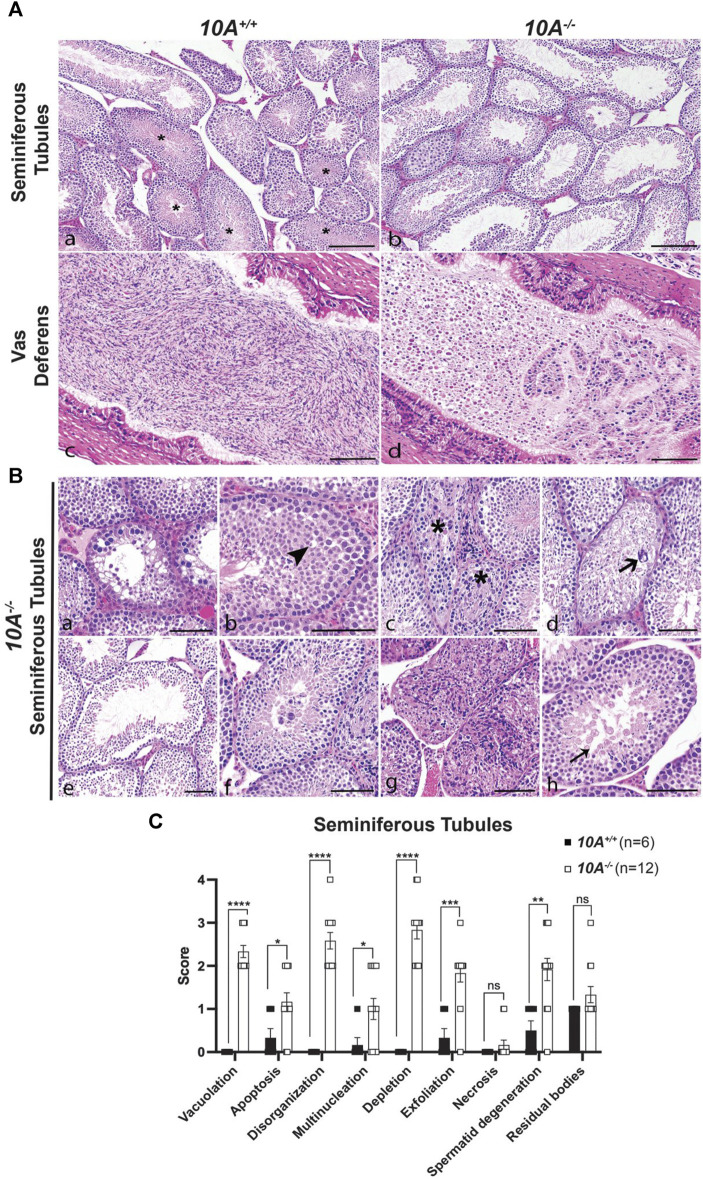
Testes and vas deferens from *Atp10A* deficient mice display several pathologic features. **(A)** Representative H&E images comparing testes (seminiferous tubules) and vas deferens between *10A* ^*−/−*^ and *10A* ^*+/+*^ mice. **(a)** WT seminiferous tubules displaying various stages of spermatogenesis, including stages VI/VII/VIII (asterisks) that contained intraluminal elongated spermatids. **(b)** Germ cell depletion in *10A* ^*−/−*^ testes lacking elongated spermatids in stages VI/VII/VIII tubules. **(c)** Vas deferens from WT mice contains large numbers of morphologically normal sperm. **(d)** Vas deferens from *10A* ^*−/−*^ mice contain mostly sloughed immature germ cells, epithelial cells, and cell debris. Scale bar = 100 μm. **(B)** Representative H&E images of pathologic features in seminiferous tubules from *10A* ^*−/−*^ mice. **(a)** Vacuolation **(b)** Apoptosis (arrowhead) **(c)** Disorganization (asterisk) **(d)**. Multinucleation (thick arrow) **(e)**. Depletion **(f)** Exfoliation **(g)** Necrosis **(h)** Spermatid degeneration (thin arrow). Scale bar = 100 μm. **(C)** Quantitative assessment of the pathological features shown in **(B)** (note that the residual bodies feature is not pictured in **(B)** (*10A* ^*+/+*^, n = 6 mice; *10A* ^*−/−*^, n = 12 mice). *p*-value by unpaired t-test (****p < 0.0001, *p = 0.0228, *p = 0.0394, ***p = 0.0003, **p = 0.0029).

To further explore the effect of *Atp10A* deficiency on male fertility, the morphology of the seminiferous tubules and the vas deferens were examined by a board-certified veterinary pathologist (TSY). Across all age groups, stages VI, VII and VIII seminiferous tubules that contain intraluminal matured, elongated spermatids were apparent from *10A* ^*+/+*^ sections (asterisks, Figure 3A, panel a), but were largely absent in the *10A* ^*−/−*^ mice (Figure 3A, panel b). Moreover, the morphology of the elongated spermatids in the seminiferous tubules from the KO mice often appeared degenerative, vacuolated, or rounded (Figure 3B, panel h). Additionally, the vas deferens from *10A* ^*−/−*^ mice displayed sloughed off immature germ and epithelial cells as well as cell debris in the tubular lumen, which were absent in *10A* ^*+/+*^ mice (Figure 3A, panel c vs. d). We scored pathologic features in the seminiferous tubules using the scoring system described in Table 1. We found seminiferous tubules from *10A* ^*−/−*^ mice displayed a significantly greater amount of vacuolation, apoptosis, disorganization, multinucleation, depletion, exfoliation, and spermatid degeneration, compared to *10A* ^*+/+*^ mice (Figures 3B,C). However, there was no difference in the amount of necrosis or residual bodies based on genotype. In addition, no difference in staining pattern for the PNA lectin acrosome marker was observed for *10A* ^*+/+*^ and *10A* ^*−/−*^ seminiferous tubules (Supplementary Figure S3C, D). Thus, developing spermatids were present within the testes of the KO mice. To further explore the morphological apoptosis phenotype observed in seminiferous tubules from the *10A* deficient mice (Figure 3C); we stained testes samples from both WT and KO mice with TUNEL stain, a marker for apoptosis. We found that *10A* deficient mice trended toward having more TUNEL staining in the seminiferous tubules compared to WT mice (Supplementary Figure S5). Taken together, *Atp10A* deficiency results in marked seminiferous tubule and vas deferens pathologies.

**TABLE 1:** Histopathologic scoring of seminiferous tubules.

Score	Approximate portion of seminiferous tubules affected (%)
0	0
1 (minimal)	<5
2 (mild)	5–25
3 (moderate)	25–50
4 (severe)	>50

A scoring system was developed to assess the severity of several different germ cell pathologies in the seminiferous tubules. The scoring system is based on the percentage of affected seminiferous tubules, with 0 representing no observable pathology and 4 indicating a severe pathologic feature observed in over 50% of the seminiferous tubules. Details about the establishment of the scoring system are outlined in Materials and Methods.

### *Atp10A* deficient mice exhibit alterations in the total amounts and relative levels of hormones regulated by the HPG-axis

**FIGURE 4:**
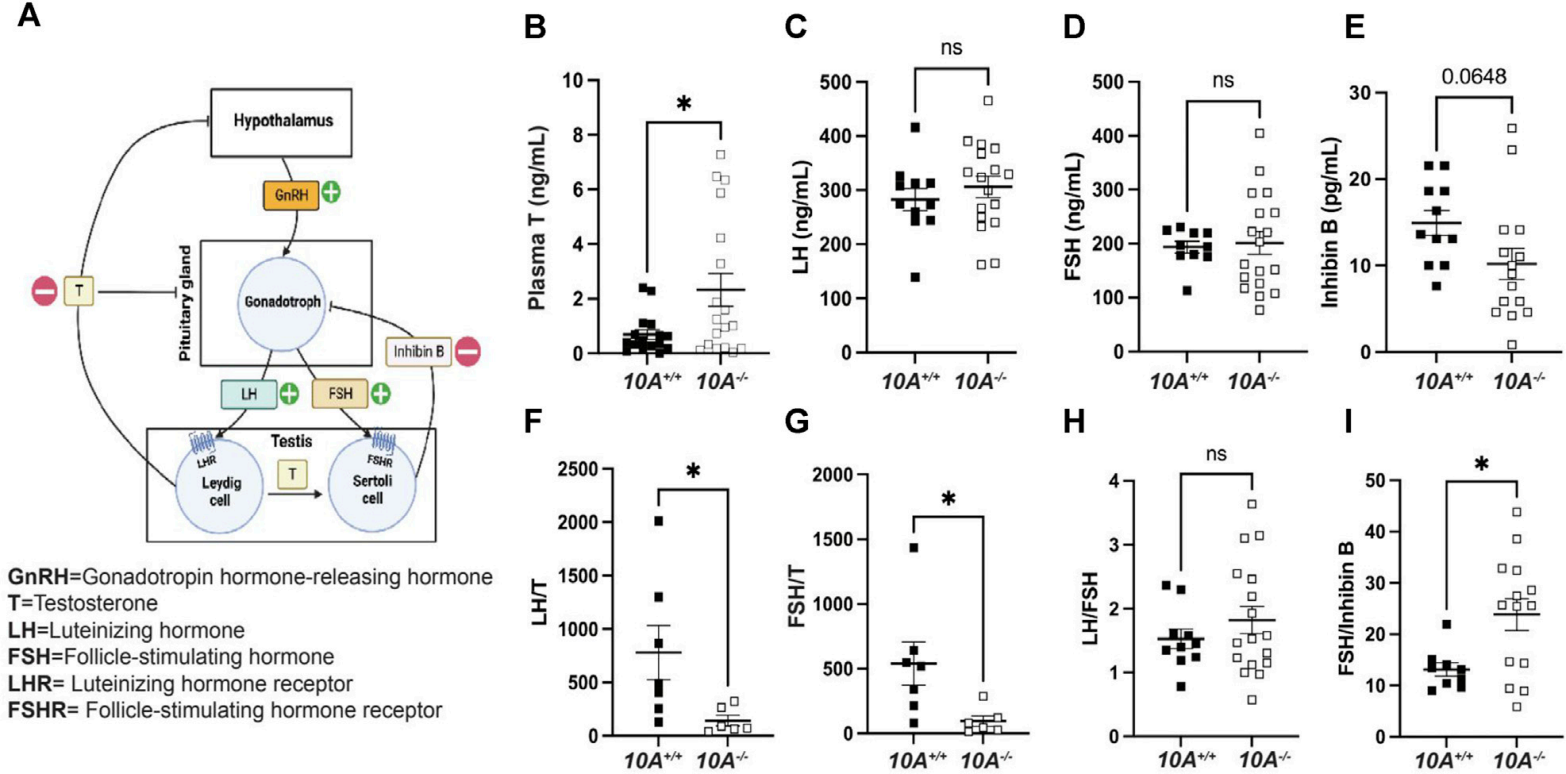
*Atp10A* deficient male mice exhibit reduced levels of circulating testosterone and altered ratios of HPG-axis-regulated hormones. **(A)** Schematic representation of the HPG-axis. Schematic created using Biorender.com. **(B–E)** ELISA analysis of circulating **(B)** testosterone (T), *p = 0.0187, **(C)** luteinizing hormone (LH), **(D)** follicle stimulating hormone (FSH), and **(E)** Inhibin B from 8–20-week-old mice. **(F–I)** Ratio of ELISA analysis of circulating **(F)** LH to T, *p = 0.0437, **(G)** FSH to T, *p = 0.0354, **(H)** LH to FSH, and **(I)** FSH to inhibin B, *p = 0.0146. *p*-value by unpaired t-test.

To further understand the mechanism of how *Atp10A* deficiency causes male infertility, we measured hormones regulated by the HPG-axis, which controls the production and release of hormones involved in male reproductive function (Figure 4A). We found that *Atp10A* deficient males have significantly higher levels of circulating testosterone (T) compared to WT littermates (Figure 4B). We did not find any significant differences in the level of circulating luteinizing hormone (LH) (Figure 4C), follicle stimulating hormone (FSH) (Figure 4D), or Inhibin B (Figure 4E), although the *Atp10A* deficient mice trended toward having lower circulating levels of Inhibin B compared to WT littermates. We also found that *Atp10A* deficient mice exhibit lower LH/T (Figure 4F) and FSH/T (Figure 4G) ratios, driven by the elevated testosterone, compared to WT mice. Additionally, *Atp10A* deficient mice exhibited no difference in their ratio of LH/FSH (Figure 4H) but did display an elevated FSH/Inhibin B ratio compared to WT mice (Figure 4I). Altogether, *Atp10A* deficiency perturbs the total levels and relative amounts of hormones regulated by the HPG-axis.

### *Atp10A* deficient mice exhibit alterations to gonadotropin-dependent signaling in the testes

**FIGURE 5:**
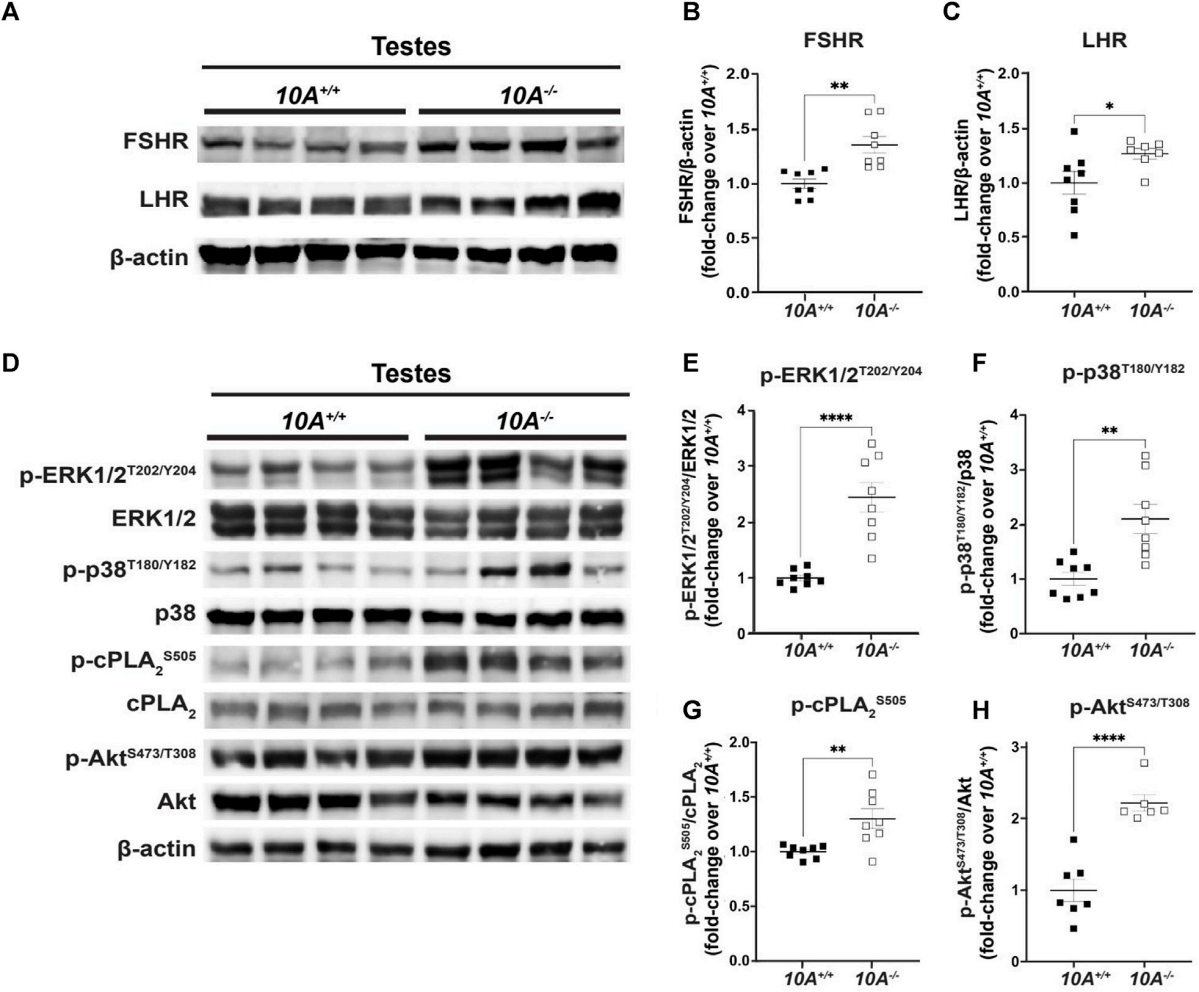
*Atp10A* deficiency results in changes to signaling pathways in the testes. Total tissue lysates from testes from the *10A^+/+^* and *10A^−/−^* mice were subjected to Western immunoblot analysis. **(A, D)** Representative blots for **(A)** total FSHR and LHR, **(D)** and total and phosphorylated ERK1/2, p38, cPLA2, and Akt. **(B, C)** For FSHR and LHR quantitation, total protein was normalized to β-actin levels and total protein from *10A^+/+^* samples. **(E, F, G, and H)** The phosphoproteins were normalized to their respective total protein, β-actin levels, and total protein from *10A^+/+^* samples. Mean measurements of 4-6 independent experiments are shown. *p*-value by unpaired t-test. (B, *10A^+/+^* n = 8, *10A^−/−^* n = 8, **p = 0.0012; C, *10A^+/+^* n = 8, *10A^−/−^* n = 7, *p = 0.0448; E,F,G, *10A^+/+^* n = 8, *10A^−/−^* n = 8, **(E)** ****p < 0.0001, (F)**p = 0.0020, (G)**p = 0.0058; H, *10A^+/+^* n = 7, *10A^−/−^* n = 6, **(H)**: ****p < 0.0001).

To explore the molecular mechanisms underlying *Atp10A* deficiency-induced infertility, we examined the levels and phosphorylation status of key proteins involved in gonadotropin signaling within the testes. First, we probed the amount of the FSH receptor (FSHR) (Casarini and Crepieux, 2019) and LH receptor (LHR) (Johnson and Jonas, 2019) and found that the *Atp10A* deficient mice exhibit elevated levels of both FSHR and LHR in their testes (Figures 5A–C). We also measured the total amount of androgen receptor (AR) (Davey and Grossmann, 2016) in testes and did not find a difference based on genotype (Supplementary Figure S6A, B). We next measured the total amounts and phosphorylation status of several proteins that have critical roles in the growth, development, and maintenance of testes, including ERK1/2, p38 MAPK (p38) and Akt. We found an increase in the activating phosphorylation of ERK1/2 at T202/Y204 (Figures 5D,E), p38 at T180/Y182 (Figures 5D,F), and Akt at S473 and T308 (Figures 5D,H) in the testes of *Atp10A* ^*−/−*^ mice. We also measured the phosphorylation state of cPLA_2_, an enzyme implicated in fertility (Bonventre et al., 1997) and previously reported by us to exhibit an altered phosphorylation state in the liver of female *Atp10A* deficient mice compared to controls (Norris et al., 2024). In the testes, we observed an increase in activating phosphorylation of cPLA_2_ at S505 in the *Atp10A* deficient mice (Figures 5D,G). We next examined the phosphorylation status and total levels of these proteins in the vas deferens and found that *Atp10A* deficient mice exhibit reduced activating phosphorylation of ERK1/2 at T202/Y204 (Supplementary Figure S7A, B) and p38 at T180/Y182 (Supplementary Figure S1A, C). We also found that the KO mice had increased activating phosphorylation of Akt at S473 and T308 (Supplementary Figure S7A, D) and cPLA_2_ at S505 (Supplementary Figure S7A, E), similar to what was observed in the testes. Taken together, *Atp10A* deficiency leads to alterations in the total amount and phosphorylation status of proteins implicated in signaling that effects the response to gonadotropins as well as growth, development, and maintenance of the testes and vas deferens.

## Discussion

In this study, we show that ATP10A protein is expressed in the vas deferens and testes of mice (Figure 1) and that *Atp10A* deficiency results in male-specific infertility characterized by small testes, oligozoospermia (due to low production or an inability for the sperm to diffuse efficiently out of the epididymal slices), and asthenozoospermia (Figure 2). Interestingly, we observed the oligozoospermia phenotype in both the caput (Figure 2D) and cauda (Figure 2F) epididymis from *10A* deficient mice; implying that *10A* deficiency impacts both immature (caput) and mature (cauda) sperm populations. Male *Atp10A* deficient mice also exhibit vas deferens and testes pathologies; including spermatid degeneration and a deficiency of stage VI-VIII seminiferous tubules with mature spermatozoa (Figure 3). Additionally, we visualized acrosomes, a specialized structure found at the tip or anterior part of the head of mature sperm cells, and at the perinuclear region of developing spermatids in seminiferous tubules, and found similar morphology and abundance of this structure in both WT and KO mice (Supplementary Figure S3C). These results suggest that ATP10A does not disrupt early stages of spermatogenesis, but most likely has a role in later stages of the sperm maturation process. We also observed alterations to total levels and relative amounts of HPG-axis controlled hormones (Figure 4), and changes to signaling in the testes (Figure 5) and vas deferens (Supplementary Figure S3).

We found that ATP10A is expressed in murine vas deferens and testes (Figure 1). There is a ubiquitous ATP10A-positive signal in the vas deferens (Figure 1C) and the lumen of seminiferous tubules of the testes (Figures 1B,D). The Mouse Cell Atlas (Wang et al., 2023) reports *Atp10A* mRNA expression in Leydig cells, Sertoli cells, peritubular cells, fibroblast, macrophages, elongated spermatid, round spermatid, spermatocytes, and differentiated spermatogonia in the testes. However, spermatogenic cells have mechanisms of delayed mRNA translation and protein expression, therefore mRNA expression in cells does not always imply protein expression (Walker et al., 1999). We detected ATP10A protein expression in the lumen of seminiferous tubules; where elongated spermatids are located (Figure 1D). However, we did not observe colocalization of the ATP10A signal with a marker for acrosomes (found at the tip/anterior part of mature sperm cells) (Supplementary Figure S3C). Therefore, ATP10A might be expressed in the sperm flagella membrane or mature spermatogonia that have their tails hanging into the lumen. No other cell type in the testes expressed sufficient ATP10A protein to be detected with available antibodies. Further work is needed to determine the precise cell type(s) or location in which ATP10A protein is expressed within the vas deferens and testes and if ATP10A is expressed in other components of the male reproductive system, such as the epididymis. Additionally, according to the Mouse Cell Atlas (Wang et al., 2023), *Atp10A* is also expressed in chondrocytes, macrophages, and several other cell types. Therefore, further investigation is required to determine if this data translates to protein expression and whether the male infertility resulting from loss of *Atp10A* is solely due to its absence in reproductive tissues or if this phenotype is also influenced by its absence in other cell types where it is expressed.

Interestingly, several members of the P-type ATPase family have roles in fertility (van der Mark et al., 2013). Two Type II P-type ATPases, Ca^2+^-ATPase isoform 4 (ATP2B4) and α -4 isoform of the Na^+^/K^+^-ATPase, are important for sperm motility (Woo et al., 2000; Prasad et al., 2004). ATP8B3 has been implicated in sperm cell acrosome formation and capacitation (Gong et al., 2024) and ATP8B5 (also known as FetA) has potential roles in spermatogenesis and acrosome biogenesis based on expression patterns (Xu et al., 2009). In this study, we show that another member of the P-type ATPase family, ATP10A, which translocates PC and GlcCer (Roland et al., 2019), has a role in the maintenance of male-specific fertility. A closely-related homolog of ATP10A, ATP10D, also translocates GlcCer (Roland et al., 2019), is transcriptionally expressed in murine (Wang et al., 2023) and human testes (Consortium, 2013; Soraggi et al., 2021), and has been implicated as being a transcript in human spermatozoa necessary for successful intrauterine insemination (Garcia-Herrero et al., 2010). Interestingly, C57BL/6J mice exhibit a stop codon in exon 12 in the *Atp10d* gene (Flamant et al., 2003), therefore the genotypes of the mice used in this study, unless otherwise noted, are *10A* ^*+/+*^ *10D* ^*−/−*^ (WT) and *10A* ^*−/−*^ *10D* ^*−/−*^ (KO). To determine any potential functional redundancy between ATP10A and ATP10D we generated *10D* ^+/+^ mice via CRISPR-mediated repair of the nonsense mutation back to the wild-type glutamine codon (Supplementary Figure S4A). We found that reintroduction of WT *Atp10D* did not rescue the infertility phenotypes in *Atp10A* deficient mice (Supplementary Figure S4B−E). Therefore, ATP10A deficiency alone is sufficient to cause the male-specific infertility phenotypes in mice and this suggests that the specific ATP10A substrate critical for male fertility might be PC and not GlcCer; because ATP10A flips both PC and GlcCer (Naito et al., 2015; Roland et al., 2019) and ATP10D only flips GlcCer (Roland et al., 2019). However, it is possible that ATP10D (and GlcCer) has a role in human fertility and this needs to be investigated.

We found that male *Atp10A* deficient mice have small testes (Figures 2B,C), oligozoospermia (Figures 2D,F) and spermatid degeneration (Figure 3C), a trend of decreased circulating levels of Inhibin B, a marker for spermatogenesis (Anderson and Sharpe, 2000), and elevated circulating levels of testosterone (Figure 4B). The combination of small testes, oligozoospermia, and elevated testosterone levels appears paradoxical, as testosterone is a critical factor in facilitating testes growth and supporting spermatogenesis (Kenneth et al., 2019). However, it is possible that the low sperm count could lead to a compensatory increase in testosterone production. Interestingly, transgenic male mice overexpressing human chorionic gonadotropin (hCG), a hormone that is structurally similar to LH that signals through the LH receptor (LHR), also exhibit small testes with elevated circulating levels of testosterone (Rulli et al., 2003). Although these disparate phenotypes co-occurring has been previously observed, the specific mechanisms remain unexplored. Reduced testicular size could persist despite elevated testosterone levels if the excessive testosterone fails to evoke the intended effects on its target tissues, including Sertoli cells, or induce the negative feedback loop in the HPG-axis (Figure 4A). To begin to probe the molecular response to the elevated testosterone in the *Atp10A* deficient mice; we measured total levels of AR in the testes, but did not find a difference based on genotype (Supplementary Figure S6A, B). Therefore, the molecular response to the elevated testosterone in *Atp10A* deficient mice remains an open and interesting question.

We focused our efforts on exploring the cause of the elevated testosterone levels in the *Atp10A* deficient mice. Elevated testosterone levels could arise from hyperactivation of LHR-mediated signaling in Leydig cells. We found that *Atp10A* deficient mice have increased levels of FSHR and LHR in the testes (Figures 5A–C). Cellular levels of FSHR and LHR are regulated through receptor endocytosis, facilitating their desensitization, or degradation of their mRNA transcripts (Krishnamurthy et al., 2003; Ulloa-Aguirre et al., 2018). The elevated levels of FSHR and LHR detected in the testes of *Atp10A* deficient mice warrants further investigation into whether these changes arise from reduced endocytosis or modifications in mRNA transcript processing. Additionally, changes to aromatase activity, the enzyme that converts testosterone to estrogen (Chan et al., 2016), could also affect levels of circulating testosterone, and this possibility should be tested.

We investigated various signaling events within the testes. In *Atp10A* deficient mice, we found increased activating phosphorylation of proteins involved in growth and control of gene expression, including p-ERK1/2, p-38 (MAPK), and Akt (Figures 5E,F,H). We also found an increase in activating phosphorylation of cPLA_2_ (Figure 5G), an enzyme whose phosphorylation state has been previously shown to be affected in *Atp10A* deficient female mice (Norris et al., 2024) and that has also been implicated in female fertility (Bonventre et al., 1997). cPLA_2_ catalyzes the hydrolysis of phospholipids, such as PC, to lysoPLs and fatty acids, such as arachidonic acid, and this fatty acid species can bind directly to GPCRs (Oka et al., 2009) or be oxidized to form eicosanoids (Niknami et al., 2009). Interestingly, we previously reported a reduction in circulating eicosanoid levels in *Atp10A* deficient female mice (Norris et al., 2024). The potential link between *Atp10A* deficiency, cPLA_2_ regulation, eicosanoid homeostasis, and fertility requires further investigation.

In the testes, Akt-dependent signaling results in multiple downstream effects (Chen et al., 2022); including facilitating proliferation and anti-apoptosis of immature Sertoli cells (Riera et al., 2012; Nascimento et al., 2016; Xi et al., 2022). There is evidence that a potent toxin produced by cyanobacteria, Microcystin-LR, which has adverse effects on reproduction; initiates Akt-dependent signaling pathways, disrupts the blood-testis barrier, and induces apoptosis in Sertoli cells (Chen et al., 2013; Zhou et al., 2018; Zhou et al., 2019; Zhou et al., 2020). Interestingly, the testes from *Atp10A* deficient mice exhibit increased apoptosis (Figure 3C, Supplementary Figure S5) and a marker of abnormal proliferation (multinucleation) as well as damage and disruption to the normal architecture of the tissue (vacuolation, disorganization, depletion, and exfoliation) (Figure 3). Whether these pathologies are caused by elevated Akt-dependent signaling in the testes (Figure 5) or defects in other processes requires further study. Furthermore, activation of LHR and FSHR in the testes can trigger phosphorylation of ERK and p38 (MAPK) (Flamant et al., 2003; Manna et al., 2009; Wang et al., 2022), as well as Akt (Carvalho et al., 2003; Fukuda et al., 2009; Riera et al., 2012; Nascimento et al., 2016; Xi et al., 2022). Understanding the relationship between the observed infertility phenotypes, receptor-specific activation of the MAPKs and Akt, and the elevated levels of FSHR and LHR in the testes in *Atp10A* deficient mice requires further study. Furthermore, detailed cell-specific differences, such as those pertaining to Leydig and Sertoli cells, cannot be elucidated at present as our measurements from the testes encompass the entirety of the organ. The exploration of cell-specific observations arising from ATP10A deficiency remains an intriguing and open question.

The testes and vas deferens are functionally interdependent components of the male reproductive system; the testes (Figure 1B) produce sperm and the vas deferens is an epithelial and smooth muscle tubule that serves as passageway for the sperm cells. ATP10A is expressed in both the testes and vas deferens (Figure 1), and these tissues exhibit similar but distinct pathologies (Figure 3) and disruptions to signaling (Figure 5, Supplementary Figure S7) in *Atp10A* deficient mice. The specific functions ATP10A has in the testes and vas deferens remains a topic for speculation, as it is yet to be determined whether the protein exerts primary influences on the testes that subsequently impact the vas deferens or *vice versa*, or if the two organs are independently affected, leading to a synergistic negative effect on fertility.

We previously showed that female *Atp10A* deficient mice fed a high fat diet exhibit dyslipidemia, but the male mice do not (Norris et al., 2024). The molecular basis of this sexual dimorphism is unknown; however, the elevated circulating testosterone levels in male *Atp10A* deficient mice may protect them against metabolic dysfunction. Indeed, testosterone deficiency in men is an independent cardiovascular risk which is associated with obesity, metabolic syndrome, and type 2 diabetes (Jones, 2010; Kelly and Jones, 2013) and testosterone treatment reverses metabolic defects in mice with low testosterone (Kelly et al., 2016). Taken together, male *Atp10A* deficient mice exhibit infertility accompanied by elevated testosterone levels, which paradoxically could confer protection against metabolic disorders.

Altogether, this study has demonstrated the importance of ATP10A in male reproductive health and its expression in both the testes and vas deferens underscores its important role in male fertility. The phenotypes caused by *Atp10A* deficiency, such as reduced testes size, oligozoospermia, asthenozoospermia, and the observed histopathological changes in the testes and vas deferens, emphasize its importance in the maintenance of male-specific fertility. Furthermore, the elevated circulating testosterone levels and changes to signaling pathways within the testes in *Atp10A* deficient mice highlight the impact ATP10A has on hormonal regulation and intracellular pathways essential for reproductive function. ATP10A translocates PC and GlcCer from the outer to the inner leaflet of the plasma membrane and further research is needed to understand how the lipid transport activity of ATP10A intersects with its role in male fertility. There is also a need to determine if mutations in *ATP10A* could be potential risk factors for male-specific infertility in humans.

## Methods

### Animals

All mouse experiments were approved under the Vanderbilt University Institutional Animal Care and Use Committee. Mice were housed in 12 h light/dark cycles in temperature and humidity-controlled facilities with ad-libitum access to diet and water. Mice in this study were sacrificed via CO_2_ euthanasia systems, operating at the recommended flow rates, followed by cervical dislocation; this method is an acceptable euthanasia method according to the American Veterinary Medical Association (AVMA) guidelines. The mouse studies described in this manuscript are reported in accordance with the ARRIVE guidelines (https://arriveguidelines.org/).

### Randomization

Experimental mice were generated using het × het breeding schemes. WT and KO mice were assigned to studies as they became available from the breeding process.

### Creating mouse models

The *Atp10A* mouse model (*Atp10Aem1(Vtrg))* and the *Atp10D* mouse model (*Atp10Dem1 (Vtrg))* were created via CRISPR-Cas9 in collaboration with the Vanderbilt Genome Editing Resource. For the *Atp10A* mouse model: Guide RNAs (crRNA) were created to target *Atp10A* on chromosome 7, exon 2 (note that exon 2 encodes a structurally essential part of the protein and there are no functional transcripts detected that skip exon 2): Target Intron 1–2: TGA​CTG​CTT​AAT​GAT​TCG​AGG, GAG​TGA​CTG​CTA​ATG​ATC​G, Target Intron 2–3: GGA​AAA​AGC​CCA​ATT​CCA​CAC, AGC​CCA​ATC​CAC​ACA​GGA​AC. Approximately 608 bp were deleted using this method: nucleotides 58389679–58390287 (NCBI reference sequence: NC_000073). More details about the creation and validation of the *Atp10A* mouse model can be found in (Norris et al., 2024). For the *Atp10D* mouse model: The purpose of the mouse model was to correct the premature stop codon in the *Atp10D* allele in C57BL/6J mice with a glutamine seen in other mouse models (X817Q). A guide RNA (crRNA) was created to target *Atp10D* on chromosome 5 (nucleotides 72203329–72298771 bp, + strand): cRNA: AGT​CAA​AGG​GCA​GAA​TGT​GT. The donor DNA sequence was: TAT​GCC​GCC​AGA​GCT​TAC​CGT​TGC​ACT​TTA​CAG​TCT​CGG​ACC​CCA​GAG​CAG​GTC​ATG​GTG​GAC​TTT​GCT​G CTT​TGG​GCT​CAT​TAA​CAT​TT C^1^AG^2^CTG^3^CTT^**4**^CAC​ATT​CTG​CCC​TTT​GAC​TCT​GTA​AGG​AAA​AGA​ATG​TCG​GTC​GTG​GTC​AGG​CAT​CCT​CTT​TCC​AAA​CAA​GTC​GTG​GTG​TAT. The superscript numbers next to the nucleotides indicate the following mutations: 1. T to C introduces the desired STOP to Q correction. 2. A to G is a silent mutation (CAA to CAG) that along with mutation 1 and 3 introduces a PvuII restriction site for screening and genotyping purposes. 3. C to G is a silent mutation (CTC to CTG = L) that along with mutation 1 and 2 introduces a PvuII restriction site and mutates the PAM in the guide RNA. 4. A to T is a silent mutation (CTA to CTT = L) that serves as a second mismatch, that along with the PAM mutation, ensures inhibition of Cas9 retargeting. The underlined sequence indicates the crRNA location. For both mouse models: CRISPR editing reagents were delivered into the pronuclei of one-cell fertilized mouse zygotes (C57BL/6J). The resulting pups were biopsied and screened by PCR and Sanger sequencing. The predicted founders were bred to WT C57BL/6J animals and the offspring were genotyped (N1 generation). The offspring with the appropriate genotype were then backcrossed two more times.

### Genotyping

Mice were genotyped using tail DNA. The *Atp10A* DNA products were detected via PCR (Q5 DNA Polymerase, NEB) followed by gel electrophoresis; *Atp10A-F* (GTG​CAC​TGT​ATT​TGT​CTG​CCT​GTT​CC), *Atp10A-R (*GGT​CCT​TTG​AAG​AGA​TAA​TGT​TCC​CAA​C). For *Atp10D* mice: *Atp10D* DNA products were detected via PCR (Q5 High-Fidelity Polymerase, NEB, catalog #M0491S) followed by gel electrophoresis; *Atp10D*-F (CAA​AAC​TGT​CAC​CTC​CTA​TGG​A), *Atp10D*-R (GTA​TAC​ACC​ACG​ACT​TGT​TTG​G) for visualization (expected band size: 567 bp). After confirming amplification of the expected target via gel electrophoresis, PCR samples were purified (QIAquick spin column (lot No. 166028242)) and sent to Genewiz (https://www.genewiz.com/) for sequencing or a restriction digest reaction was done (PvuII-HF enzyme (NEB, catalog #R3151S), followed by gel electrophoresis and visualization. PCR products from *Atp10D* mice without the X817Q allele were not cut during the restriction digest (1 band on the gel (567 bp)) and those with the X817Q allele were cut (2 bands on the gel (567 bp, ∼400 bp)).

### Generation of ATP10A antibodies

Anti-ATP10A antibodies were produced by Vanderbilt Antibody and Protein Resource. ATP10A peptides 1 (27 RTRTVRSNLLPPC 38), 3 (498 HKTQSIKSHRRTC 510) and 5 (1,280 QTLLGDPLFYLTC 1292) for antibody production were chosen based on conservation between mouse and human ATP10A orthologs, lack of conservation with ATP10B and ATP10D paralogs, and predicted water solubility (https://pepcalc.com) (Supplementary Figure S1A, B). Peptide epitopes were synthesized (GenScript, United States, Inc.) with a C-terminal Cys residue and conjugated to Imject™ Maleimide-Activated mcKLH, Imject™ Maleimide-Activated BSA, and SulfoLink™ Coupling Resin following the manufacturer’s recommended protocol (ThermoFisher Scientific) by the Vanderbilt Antibody and Protein Resource (VAPR) group. The three peptide-Keyhole Limpet Hemocyanin (KLH) conjugates were used for antibody production in a single rabbit (VU579, Cocalico, Stevens, PA). Antibodies recognizing the peptide epitopes were purified from the rabbit by differential affinity chromatography using the peptide conjugated resin. Briefly, 5 mL of antisera was passed over the peptide 1 column (2 mL) and the material that failed to bind was then applied sequentially to the peptide 3 and 5 columns. After washing with 40–50 mL PBS, bound antibodies were eluted from each column with 8.5 mL of 100 mM glycine, pH 2.5 and collected in tubes containing 1.5 M Tris-HCl, pH 8. Finally, to ensure peptide specificity, antibodies eluted from one peptide column were applied sequentially to the other two columns and the flow-through was retained. Affinity purifications were performed in duplicate to generate anti-peptide 1A and 1B, anti-peptide 3A and 3B, and anti-peptide 5A and 5B antibodies (only 1 replicate is shown in Supplementary Figure S1C).

### Cell cultures and immunoblot analysis for assessment of specificity of anti-ATP10A antibodies

WT HeLa cells (WT) or HeLa cells that were transfected with an HA-tagged human ATP10A overexpression vector (HA-ATP10A-OE; a generous gift from Dr. Hye-Won Shin) were used to assess the specificity of the affinity purified anti-ATP10A antibodies. Briefly, WT and HA-ATP10A-OE HeLa cells were maintained in growth medium, DMEM with 10% FBS and 1% penicillin/streptomycin (Life Technologies, Gaithersburg, MD), at 37°C with 5% CO_2_. For immunoblot analysis, cells were cultured in growth medium in 100 mm cell culture plates to 90%–100% confluency. Cells were washed with PBS and total protein extraction was performed using M-PER reagent (Thermo Scientific, Waltham, MA, United States) with phosphatase (Sigma-Aldrich, P8340, St. Louis, MO, United States) and phosphatase inhibitor cocktails 1 and 2 (Sigma-Aldrich, P5726 and P0044, respectively). Protein concentration was quantified using BCA Reagent (Pierce, Rockford, IL). Protein extracts (40 μg) were subjected to Western immunoblot analysis using anti-ATP10A peptides 1, 2 and 3 antibodies (1:200 dilution). Anti-HA antibody (catalog #3274S, Cell Signaling) were used as a positive control. Immunoblots were developed using the Western Lightning Chemiluminescence Plus detection system (PerkinElmer, Wellesley, MA) according to the manufacturer’s protocol. Images of the immunoblot bands were obtained using AI600 CCD Imager for chemiluminescent assays (Amersham). Only anti-peptide 1 recognized full-length ATP10A (Supplementary Figure S1C). Anti-peptide 1 antibodies were used in this study.

### Tissue collection and immunoblot analysis

Western immunoblots were initially performed and quantified blinded to the genotype. After unblinding, samples were grouped by genotype and re-run for presentation of the data. Testes and vas deferens tissues were collected from mice after CO_2_ sacrifice and cervical dislocation, flash frozen in liquid nitrogen and then kept at −80°C until further processing. For immunoblot analysis, tissues were then weighed and lysed in T-PER reagent (Thermo Scientific, Waltham, MA, United States) with protease inhibitors (Sigma-Aldrich, P8340, St. Louis, MO, United States) and phosphatase inhibitor cocktails 1 and 2 (Sigma-Aldrich, P5726 and P0044, respectively), using a Polytron homogenizer. Protein concentration was quantified using BCA Reagent (Pierce, Rockford, IL). Protein extracts (100 μg) were subjected to Western immunoblot analysis. The following primary antibodies were used for detection of: ATP10A (Vanderbilt Antibody and Protein Resource, Vanderbilt University; 1:200), FSHR (LS-C331489/121448, LSBio; 1:1,000), LHCGR/LHR (LS-C334599/229916, LSBio; 1:1,000), phospho-ERK1/2^T202/Y204^ (#4370, Cell Signaling; 1:10,000), ERK1/2 (#4695, Cell Signaling; 1:5,000), phospho-p38^T180/Y182^ (#4511, Cell signaling, 1:5,000), p38 (#9212, Cell Signaling; 1:5,000), phospho-Akt^T308/S473^ (#13038/#4060, Cell Signaling; 1:1,000), Akt (#9272, Cell Signaling; 1:1,000), phospho-cPLA_2_α^S505^ (#53044, Cell Signaling; 1:1,000), cPLA_2_α (#5249, Cell Signaling; 1:1,000), AR (Abcam, ab133273), 1:1,000). Antibody to β-actin (#3700, Cell Signaling; 1:10,000) was used to evaluate protein loading in each lane. Immunoblots were developed using the Western Lightning Chemiluminescence Plus detection system (PerkinElmer, Wellesley, MA) according to the manufacturer’s protocol. Note that boiling of yeast samples caused aggregation of Drs2p (a yeast homolog of ATP10A) such that the protein would remain in the stacking gel during SDS-PAGE (Chen et al., 1999). Images of the immunoblot bands were obtained using AI600 CCD Imager for chemiluminescent assays (Amersham). Densitometry of the immunoblot bands was performed using ImageJ. For quantification, OD of bands for phosphoprotein was normalized to total protein after normalization to β-actin; otherwise, OD of bands for total protein was normalized to β-actin. Fold-change over mean value of *10A* ^*+/+*^ samples was calculated for graphs.

### Immunofluorescent staining and imaging of mouse tissues

Testes and vas deferens were collected from mice after CO_2_ sacrifice and cervical dislocation. Vas deferens samples were fixed in 10% neutral buffered formalin and testes samples were fixed in Modified Davidson’s fixative (Polysciences, catalog #24355–500) and further processing and paraffin embedding was done by the Vanderbilt Translation Pathology Shared Resource (TPSR). Tissue sections (5 μm thick) underwent re-hydration, antigen retrieval, blocking, and antibody treatment. The slides were treated with rabbit anti-ATP10A (1:20) and Lectin PNA conjugated to Alexa Fluor 568 (1:500, ThermoFisher Scientific, catalog #L32458); followed by goat anti-rabbit Alexa-Fluor 488 secondary antibody (1:500, Abcam, #150077). Slides were mounted using DAPI Fluoromount-G (Southern Biotech, catalog # 0100-20). Images of tissues were acquired using a confocal microscope (Zeiss LSM 880 with AiryScan) with a Zeiss C Plan Apochromat ×40/1.40 Plan-Apochromat Oil using the ZEN black software. Laser irradiation at 561 nm was used to excite Alexa-Fluor 568, at 488 nm to excite Alexa-Fluor 488, and at 405 nm to excite DAPI. Detector gain and laser intensity were constant for all experimental groups. Images were edited using ImageJ (images from the same tissues were edited using the same scripts). To determine background staining; the same ImageJ script was applied to the images to color them grey, apply a threshold, and calculate a gray value (arbitrary unit); after this process, the remaining signal found in KO tissues was considered nonspecific background staining; indicated by white asterisks (Figure 1D, Supplementary Figure S3C) or red, blue, and yellow boxes (Supplementary Figure S3A). Manders’ coefficient was calculated using the ImageJ plugin JaCoP (Bolte and Cordelieres, 2006).

### Mating studies

Monogamous mating pairs (1 male and 1 female) with the reported genotypes were housed together for 6–21 weeks and the frequency of litters and number of pups born in each litter were recorded (see Supplementary Table S1 for details). Various mating times were employed to obtain an average number of litters produced while minimizing the need for sacrificing a large number of pups.

### Weighing body mass and testes

Researchers were blind to the genotype of the mice during the following procedures. For measuring body mass: the body mass of the mice was measured by placing a live mouse on a scale. For weighing testes: mice were sacrificed using CO_2_ euthanasia systems followed by cervical dislocation and testes were removed from mice, and the wet mass was weighed (both testes together) using an analytical scale.

### Evaluation of epididymal sperm

Mice were housed alone for 1 week prior to sperm counts to avoid any social subordination effects. Researchers were blind to the genotype of the mice during the following procedure: mice were sacrificed using CO_2_ euthanasia systems followed by cervical dislocation, and the caput or cauda epididymis was removed from mice, cut into several pieces (to release the sperm) and then incubated in 1X PBS at 37°C for 10 min. After the incubation; 10 µL of the sperm solution was added to a hemocytometer (Petroff-Hausser Counter, Hausser Scientific, Catalog #3900), and the sperm cells were viewed and counted using the Inverted Tissue Culture Microscope with 5 MP Digital Camera (AmScope, catalog # IN200TB-5 MA). The percent of motile sperm was calculated by dividing the sperm that were moving by the total sperm cells counted.

### Histopathologic assessment of testes and vas deferens

Testes and vas deferens were collected from mice after euthanasia via CO_2_ followed by cervical dislocation. The tissues were fixed in 10% neutral buffered formalin for 48 h before undergoing further processing, paraffin embedding, and sectioning done by the Vanderbilt Translation Pathology Shared Resource (TPSR). Tissue sections (5 μm thick) were stained with hematoxylin and eosin (H&E). A board-certified veterinary pathologist (TSY) was blind to the genotype of the mice and scores were assigned to each pair of testes according to a modified scoring system (Table 1) established based on the presence and extent of pathologic features (Lanning et al., 2002; Creasy et al., 2012). Vas deferens were examined separately and not scored.

### TUNEL Assay

Slides were placed on the Leica Bond RX IHC stainer. All steps besides dehydration, clearing and cover slipping were performed on the Bond RX in TPSR. Slides were deparaffinized. Antigen retrieval was performed on the Bond RX using Triton X-100 (Cat#T9284, St. Louis, MO) for 5 min. Slides were incubated with Equilibration Buffer (#G7130, Promega, Madison, WI) for 5 min, followed with the TdT reaction mix (#G7130, Promega, Madison, WI) for 10 min, and SSC-x20 (#G7130, Promega, Madison, WI) for 10 min. The Bond Intense R detection system (#DS9263, Leica, Buffalo Grove, IL) was used for visualization. Slides were dehydrated, cleared and cover slipped. A defined surface area of 8.15 mm^2^ that contained approximately 50–60 seminiferous tubules from each section was randomly selected for analysis of the TUNEL assay. Positive cell detection and counting was performed using QuPath open-source software for digital image analysis (Bankhead et al., 2017). Two serial sections per sample were analyzed and the positive cell counting results (as a percentage of total cells counted) were recorded.

### Measurement of circulating hormones

Mice were sacrificed using CO_2_ euthanasia systems followed by cervical dislocation, and blood was collected via cardiac puncture and put into a tube with 5 µL of 0.5M EDTA. After 20 min of centrifugation at 1,000 *× g* at 4°C the plasma was collected and stored at −20°C before analysis. Total testosterone, LH, FSH, and Inhibin B levels were measured using ELISA kits (Testosterone: CrystalChem, catalog #80552, LH: MyBioSource, catalog # MBS2514287, FSH: MyBioSource, catalog # MBS2700327, Inhibin B: MyBioSource, catalog # MBS2088142). Ratios were calculated by dividing the total levels of the hormones measured from the same mouse.

### Statistics

All statistical analysis was done using GraphPad Prism, version 9.5.0 (GraphPad Software). Error bars indicate mean with standard error of the mean (SEM). When one factor with three or more groups was compared, an Ordinary one-way ANOVA was performed with Dunnett’s correction for multiple comparisons. When more than 2 factors were compared, a 2-way ANOVA was used with a Sidák’s correction for multiple comparisons. Differences between group mean values were tested using a 2-tailed Student’s *t* test. A *p*-value of less than 0.05 was considered statistically significant.

### Study approval

The animal protocol (protocol #M2000034-01) was approved by Vanderbilt University Medical Center and IACUC and all methods were performed in accordance with the relevant guidelines and regulations.

## Data Availability

The original contributions presented in the study are included in the article/Supplementary Material, further inquiries can be directed to the corresponding author.

## Supplementary material

SUPPLEMENTARY FIGURE S1 Validation of *ATP10A* antibody: expression of *ATP10A* in HeLa cells with over-expressed *HA-ATP10A*. **(A)** Alignments of human (h), rat (r), and mouse (m) *ATP10A*, *ATP10B*, and *ATP10D* created using Clustal Omega with the *ATP10A* peptides that were chosen for antibody production highlighted. These peptides were chosen based on conservation between mouse and human *ATP10A* orthologs and lack of conservation with *ATP10B* and *ATP10D* paralogs. **(B)** Table indicating the sequences of the *ATP10A* peptides that were injected into rabbits to produce antibodies, peptides are color-coded according to the colors of the peptides in **(A)**. **(C,D)** Representative images of total cell lysates (40 µg total protein) from HeLa cells with (+) or without (-) over-expressed (OE) human *HA-ATP10A* that were subjected to Western immunoblot analyses for expression of *ATP10A* using antibody produced against **(C)** *ATP10A* peptides or **(D)** HA. Peptides are color-coded according to the colors of the peptides in **(A)**.

SUPPLEMENTARY FIGURE S2 Images of reproductive organs from *10A^+/+^* and *10A^−/−^* male mice. The reproductive organs were photographed immediately after removal from 11-week old mice. The asterisk indicates a bladder filled with urine. Scale bar = 1 cm. Epidid. = epididymis.

SUPPLEMENTARY FIGURE S3 Measuring fluorescence intensity and colocalization in testes IF images. **(A)** Representative IF images of testes from *10A^+/+^* and *10A^−/−^* male mice (shown in Figure 1D) were edited and **(B)** an arbitrary grey value (representing fluorescence intensity) was calculated for each image using ImageJ (see Materials and Methods for more details) (*10A^+/+^*, n = 9 images analyzed from 2 mice; *10A^−/−^*, n = 6 images analyzed from 2 mice). P-value by unpaired t-test (*P=0.0126). **(C)** Representative immunofluorescent images of testes from *10A^+/+^* and *10A^−/−^* mice showing staining of *ATP10A* (green), Lectin (yellow), and DAPI (blue). Average Manders’ coefficient for Lectin and *10A* overlap in images from WT mice: 0.033. White asterisks indicate background staining. Scale bars= 40 µm. (*10A^+/+^* n = 6 images analyzed from 2 mice,*10A^−/−^* n = 8 images analyzed from 2 mice). **(D)** Representative immunofluorescent images of testes from *10A^+/+^* and *10A^−/−^* mice showing staining of lectin (yellow). Scale bars= 40 µm.

SUPPLEMENTARY FIGURE S4 Reintroduction of *Atp10D* does not rescue infertility, smaller testes, or low sperm cell count in *Atp10A* deficient mice. **(A)** Sequencing was performed to validate the genotype of the *Atp10D* mice. Briefly, CRISPR was used to replace the premature stop codon found in the *Atp10D* sequence in C57BL/6J mice (*, *Atp10D^−/−^* mice) to glutamine that is found in other mouse strains (Q, *Atp10D^+/+^* (corrected) mice). The underlined nucleotides indicate CRISPR-mediated changes to the DNA sequence, for the purposes of changing the amino acid or introducing restriction digest cut sites for genotyping purposes. **(B)** Mating studies were done by housing pairs of mice together (without separating) for approximately 9 weeks and recording the number of litters produced. (See supplemental table 1 for more details). **(C)** The wet mass of testes was measured in 14-21-week-old mice and was normalized to body mass of the mice, *P=0.0432. (See Supplemental Table 1 for the unadjusted testes mass and body mass values). **(D)** Sperm cells collected from the caput epididymis were counted using a hemocytometer, ****P<0.0001. **(E)** % Sperm cell motility was measured using the equation: motile sperm/total sperm counted. P-value by unpaired t-test.

SUPPLEMENTARY FIGURE S5
*Atp10A* deficient mice trend toward having more TUNEL staining in seminiferous tubules compared to WT mice. **(A)** Representative photomicrographs of TUNEL assay of seminiferous tubules showing increased apoptotic germ cells (brown nuclear and/or intracytoplasmic labeling) in *10A^−/−^* mouse testes. Scale bar = 100 μm. **(B)** Quantitation of TUNEL assay results (details in Materials and Methods) (*10A^+/+^* n = 6 serial sections measurements from 3 mice,*10A^−/−^* 6 serial section measurements from 3 mice). P-value by unpaired t-test.

SUPPLEMENTARY FIGURE S6 Androgen receptor expression is similar in total testes lysates from *10A^+/+^* and *10A^−/−^* mice. Total tissue lysates from testes from the *10A^+/+^* and *10A^−/−^* mice were subjected to Western immunoblot analysis. **(A)** Representative blots for total androgen receptor (AR), total and phosphorylated Akt. β-actin was used as a loading control. **(B)** For AR quantitation, total protein was normalized to β-actin levels. **(C)** p-Akt was normalized to total Akt after normalization to β-actin levels. **(B, C)** Fold-change over mean value of *10A^+/+^* samples was calculated. Mean measurements of 3 independent experiments are shown. P value by unpaired t-test. (B,*10A^+/+^* n=9, *10A^−/−^* n=10; C, *10A^+/+^* n=3, *10A^−/−^* n=3, **P=0.0057). Note that a subset of the mice used for the **(B)** AR measurements were probed for **(C)** p-Akt levels to demonstrate that these mice exhibited analogous signaling outcomes as those depicted in Figure 5.

SUPPLEMENTARY FIGURE S7
*Atp10A* deficiency results in changes to signaling pathways in the vas deferens. Total tissue lysates from vas deferens from the *10A^+/+^* and *10A^−/−^* mice were subjected to Western immunoblot analysis. **(A)** Representative blots for total and phosphorylated ERK1/2, p38, Akt, and cPLA2. **(B−E)** The phosphoproteins were normalized to their respective total protein, β-actin levels, and total protein from *10A^+/+^* samples. Mean measurements of 4-6 independent experiments are shown. P value by unpaired t-test. (*10A^+/+^* n = 7, *10A^−/−^* n = 7, **(B)**: *P = 0.0313, **(C)**: **P = 0.0024, **(D)**: ****P < 0.0001, **(E)**: *P = 0.0376).

SUPPLEMENTARY TABLE S1 Characteristics of mice used in this study. The table includes information on sex, sample size, age and body mass at time of sacrifice, mating study information, and statistical outlier testing.
