# Supplementary figures and images for: ATP10A deficiency results in male-specific infertility in mice
Source: Front Cell Dev Biol. 2024 Feb 13;12:1310593. doi: 10.3389/fcell.2024.1310593 (PMC10896839; doi:10.3389/fcell.2024.1310593)

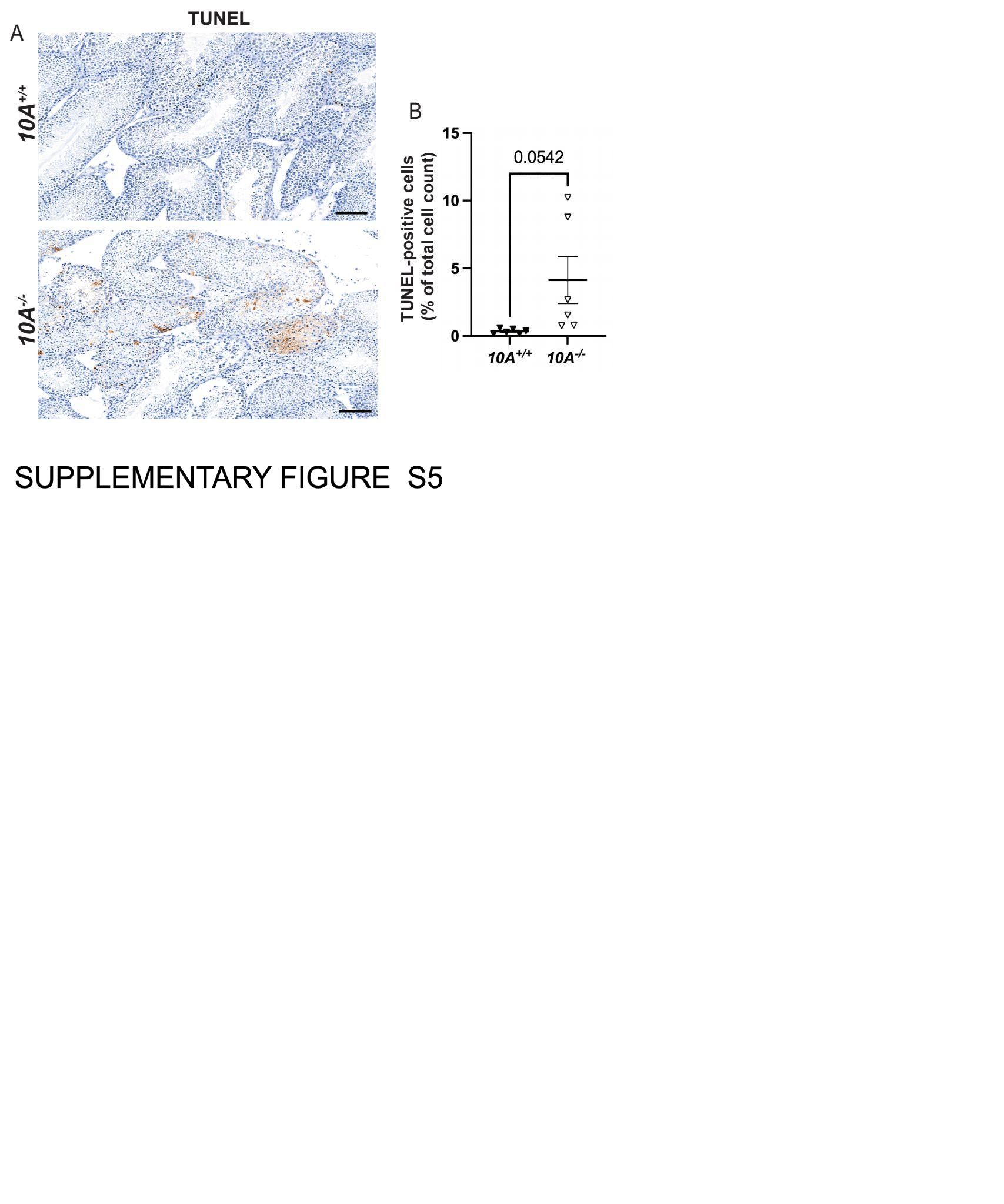

Supplement: Supplementary file 2 [file Image5.jpg]

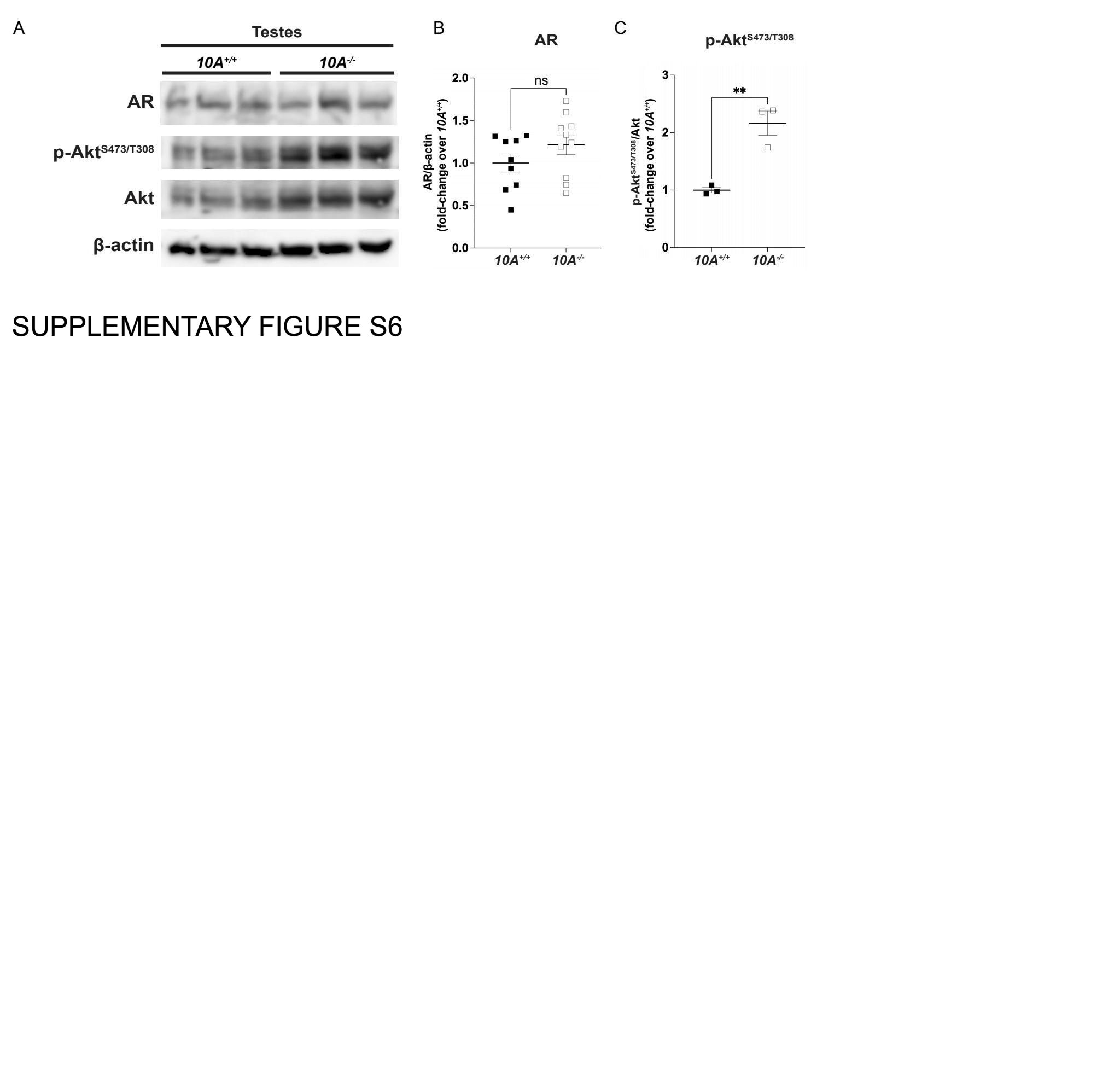

Supplement: Supplementary file 3 [file Image6.jpg]

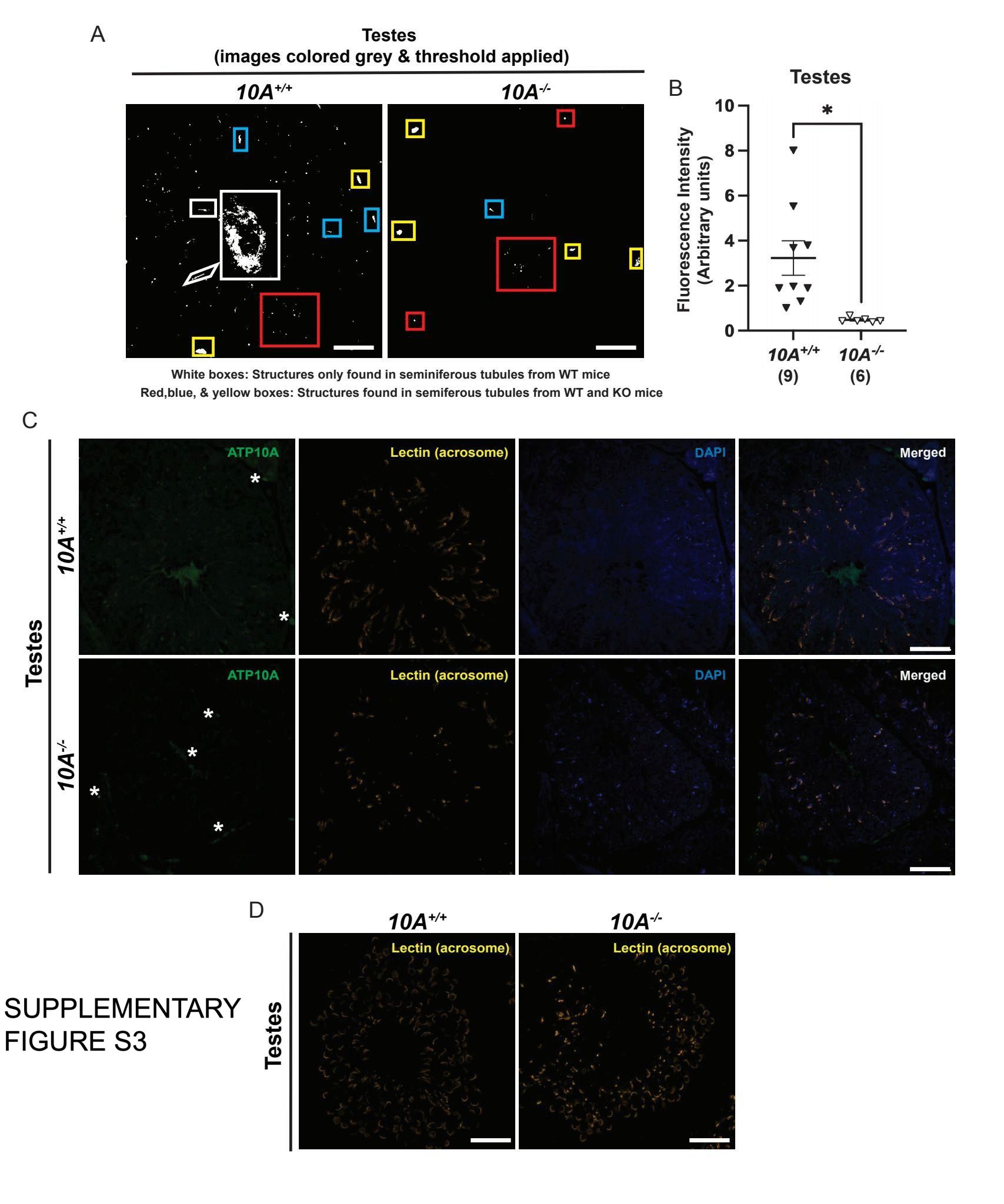

Supplement: Supplementary file 4 [file Image3.jpg]

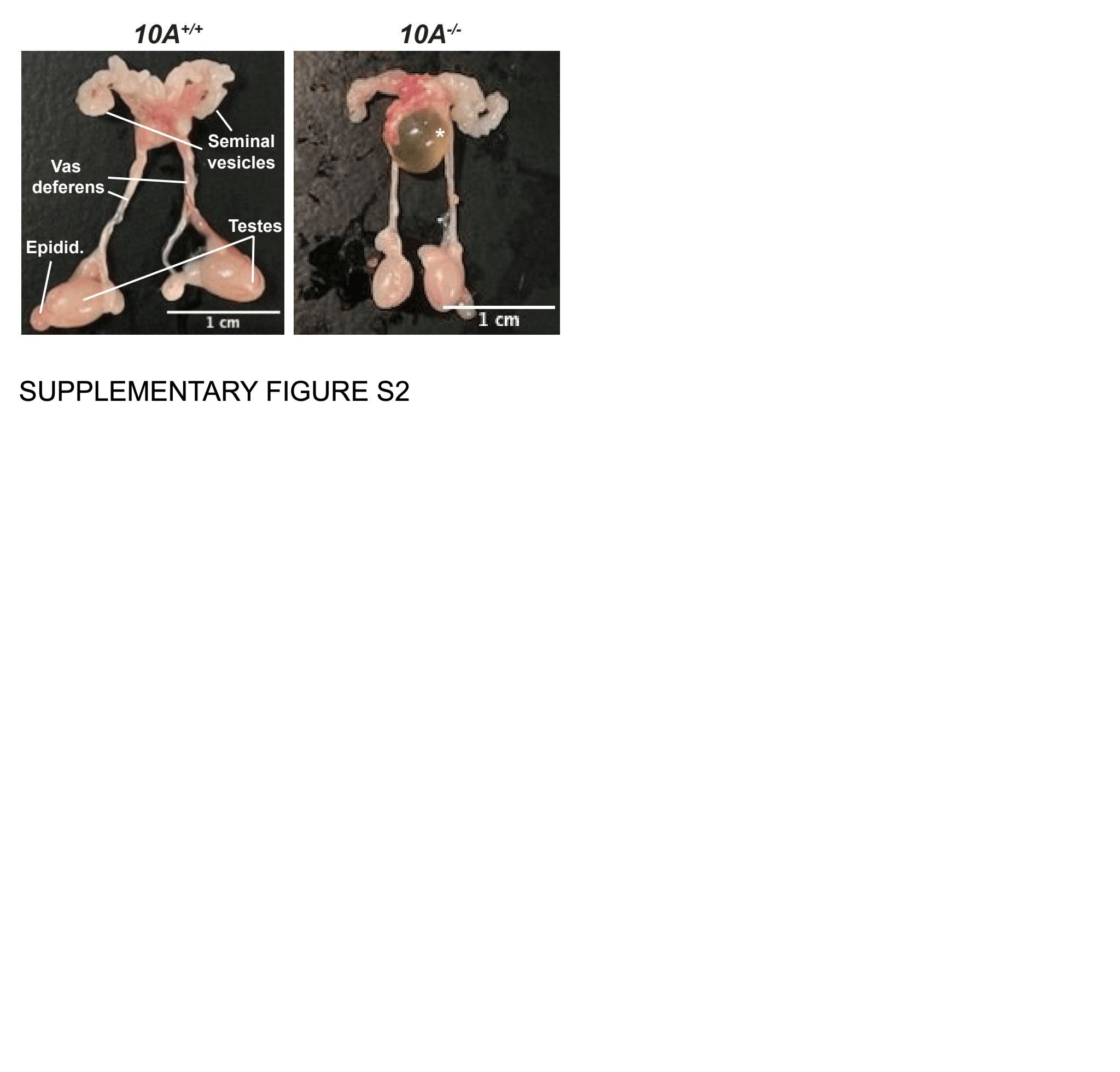

Supplement: Supplementary file 5 [file Image2.jpg]

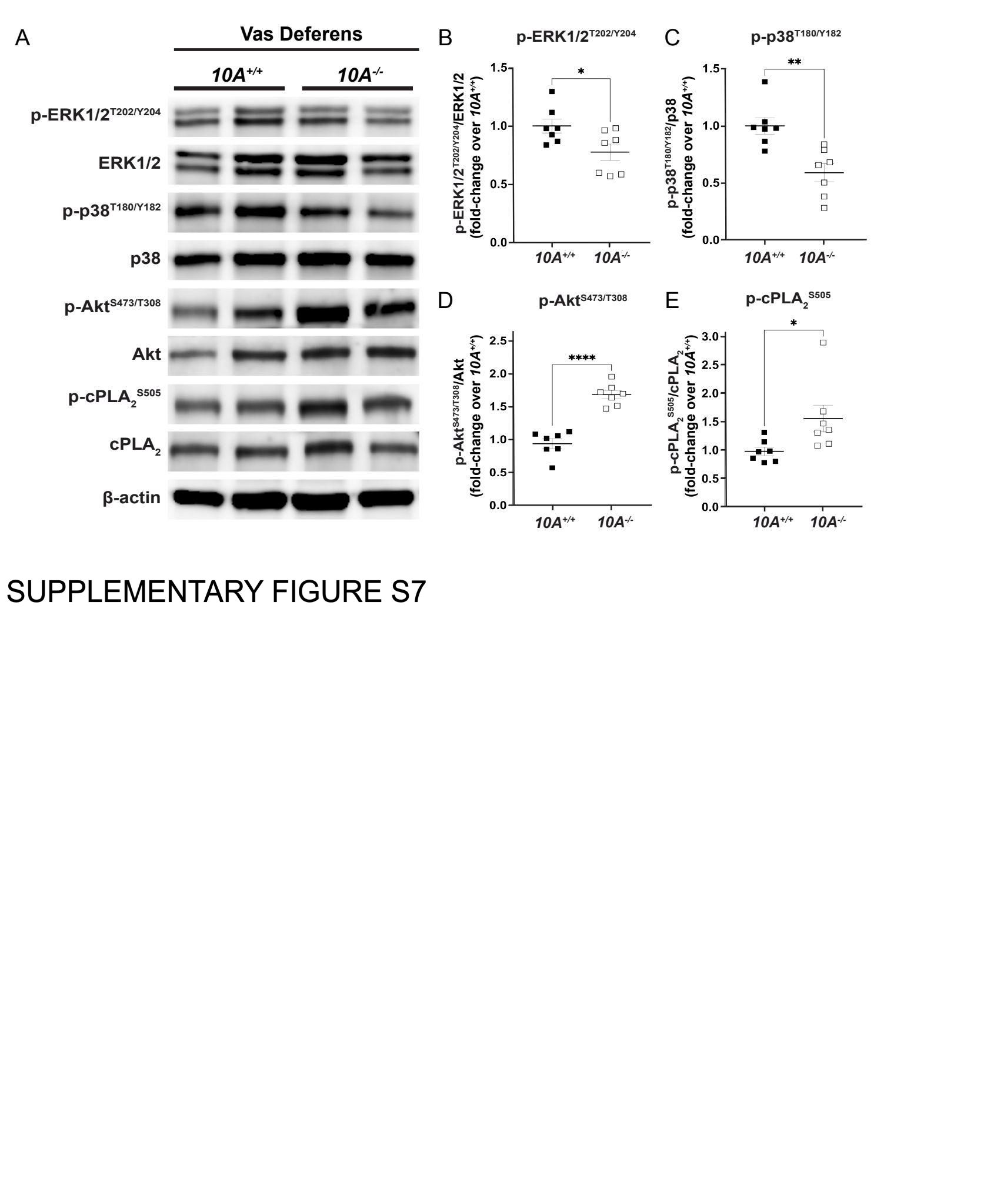

Supplement: Supplementary file 6 [file Image7.jpg]

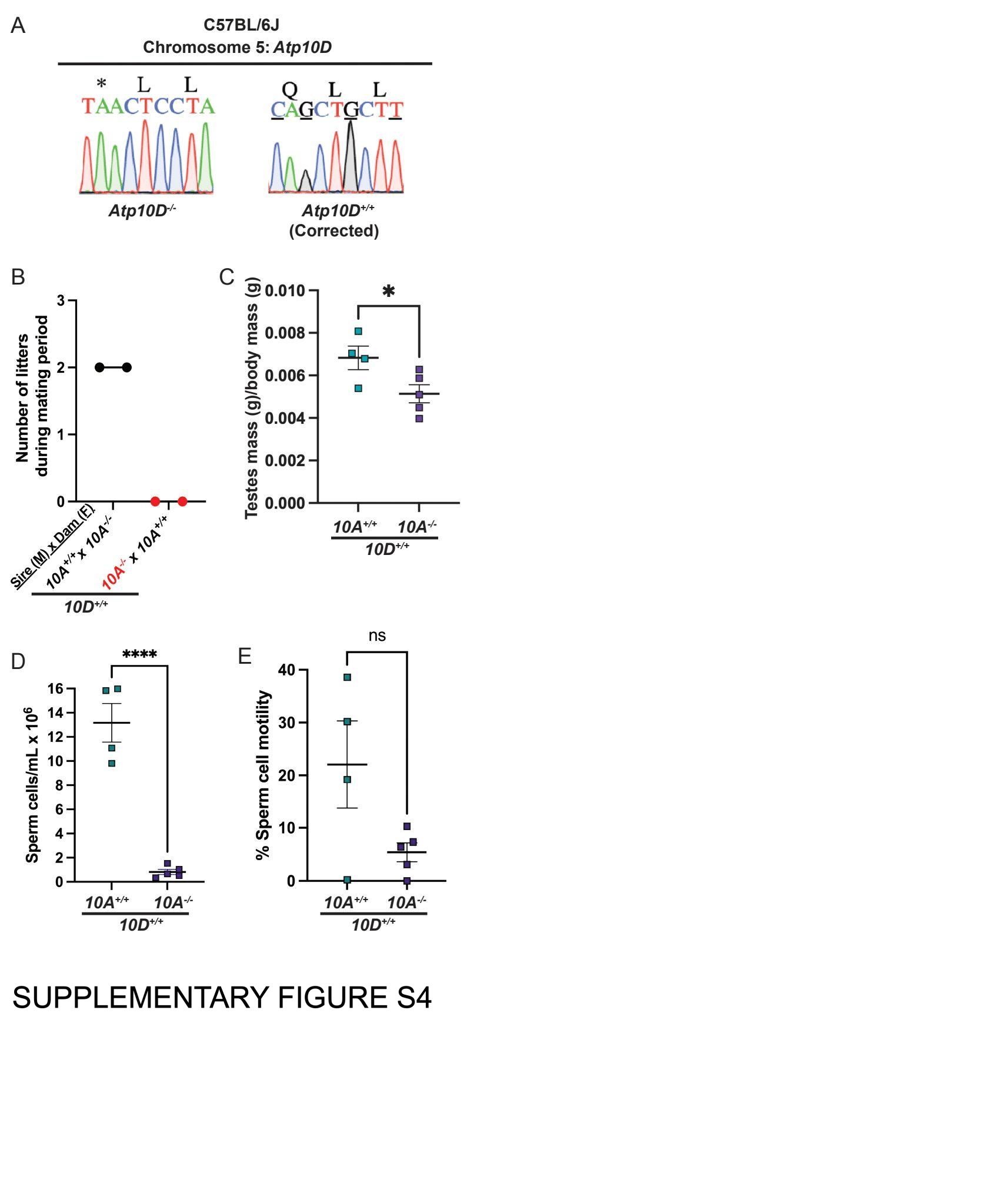

Supplement: Supplementary file 8 [file Image4.jpg]

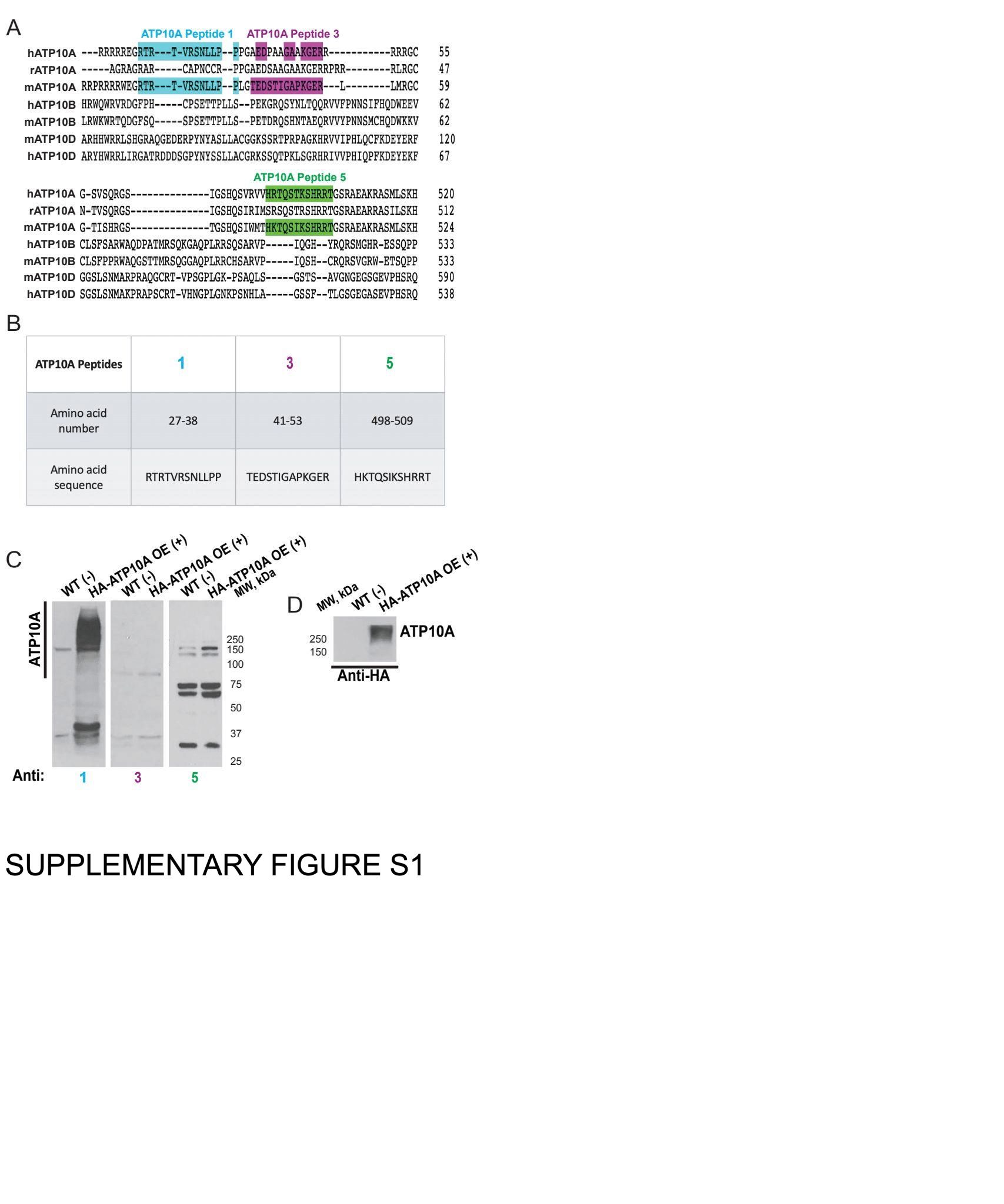

Supplement: Supplementary file 9 [file Image1.jpg]
